# Area-Efficient Integrated Current-Reuse Feedback Amplifier for Wake-Up Receivers in Wireless Sensor Network Applications

**DOI:** 10.3390/s22041662

**Published:** 2022-02-21

**Authors:** David Galante-Sempere, Dailos Ramos-Valido, Sunil Lalchand Khemchandani, Javier del Pino

**Affiliations:** Institute for Applied Microelectronics (IUMA), Department of Electronics and Automatic Engineering, University of Las Palmas de Gran Canaria (ULPGC), Campus Universitario de Tafira, 35017 Las Palmas de Gran Canaria, Spain; dramos@iuma.ulpgc.es (D.R.-V.); sunil@iuma.ulpgc.es (S.L.K.); jpino@iuma.ulpgc.es (J.d.P.)

**Keywords:** wake-up receiver, radiofrequency envelope detector, tuned radiofrequency, low power, energy efficient, wireless sensor network, complementary metal-oxide semiconductor

## Abstract

Wireless sensor network (WSN) applications are under extensive research and development due to the need to interconnect devices with each other. To reduce latency while keeping very low power consumption, the implementation of a wake-up receiver (WuR) is of particular interest. In WuR implementations, meeting high performance metrics is a design challenge, and the obtention of high-sensitivity, high data rate, low-power-consumption WuRs is not a straightforward procedure. The focus of our proposals is centered on power consumption and area reduction to provide high integrability and maintain a low cost-per-node, while we simultaneously improve circuit sensitivity. Firstly, we present a two-stage design based on a feedback technique and improve the area use, power consumption and sensitivity of the circuit by adding a current-reuse approach. The first solution is composed of a feedback amplifier, two op-amps plus a low-pass filter. The circuit achieves a sensitivity of –63.2 dBm with a power consumption of 6.77 µA and an area as low as 398 × 266 µm^2^. With the current-reuse feedback amplifier, the power consumption is halved in the second circuit (resulting in 3.63 µA), and the resulting circuit area is as low as 262 × 262 µm^2^. Thanks to the nature of the circuit, the sensitivity is improved to –75 dBm. This latter proposal is particularly suitable in applications where a fully integrated WuR is desired, providing a reasonable sensitivity with a low power consumption and a very low die footprint, therefore facilitating integration with other components of the WSN node. A thorough discussion of the most relevant state-of-the-art solutions is presented, too, and the two developed solutions are compared to the most relevant contributions available in the literature.

## 1. Introduction

WSN applications are under extensive research and development due to the need to interconnect devices with each other. Both industry and academia have shown an increasing interest in promoting internet of things (IoT) and internet of everything (IoE) devices to boost connectivity and enable complex, coordinated tasks. A WSN can be constructed by several low-cost, low-power wireless devices characterized by a simplistic structure. They also require a very low level of human interaction for maintenance. In this sense, a WSN node is designed with a long lifespan, as in many situations, the nodes are under critical environmental conditions and the batteries are hard to replace. Conventional radios play a crucial role in WSN nodes’ operation since they are frequently one of the most expensive components in terms of power consumption [1]. A common solution to obtain significant power savings consists in duty cycling the radio interface [2]; this is applied by turning it off and on again to save power using hibernation or sleep modes, or using it only when necessary, as defined by the unscheduled IEEE 802.11 communications protocol. The most severe drawback of such an approach is that the sensor node stays unreachable and unable to communicate during the times when its radio interface is in sleep mode, which may translate to a significant latency increment. However, depending on the network’s activity, two different applications can be recognized: low-activity applications with a low-average throughput (such as WSNs for area monitoring, habitat monitoring, industrial monitoring, environmental sensing or threat detection, which consist of monitoring rare events and therefore do not require constant operation), and high-activity applications with a high-average throughput (such as multimedia and mobile WSNs, which require almost constant operation, high bandwidth, data processing and compressing techniques). In low-throughput applications, the sensor nodes’ key metrics are sensitivity and power consumption, because the sensor is expected to be placed in a harsh environment and should present a wide lifespan but with reasonable sensitivity, or it would be unreachable. On the other hand, in high-throughput applications, the latency and data rate are critical, as the node is expected to exchange a high amount of information and should respond quickly to any queries the other nodes may produce. In this context, duty cycling the radio interface severely damages the overall network performance, and many applications may not support the associated delays.

**Figure 1:**
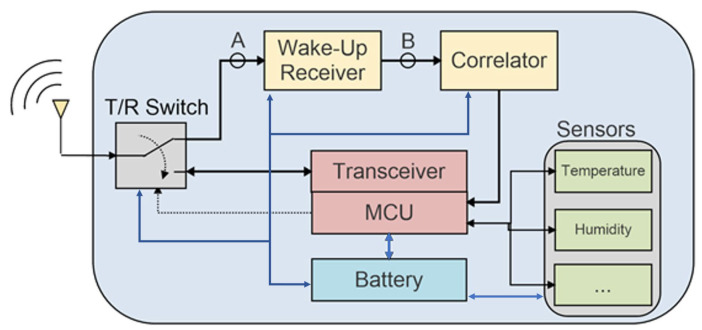
Block diagram of a sensor node integrating a WuR [3].

A well-known approach to reduce latency while keeping a very low power consumption consists in the implementation of a WuR, so that the WSN node is listening to the communication channel and the main microcontroller unit (MCU), and conventional radio communication modules are in sleep mode. A block diagram of a WSN node containing a WuR is depicted in Figure 1 [3].

The MCU and the conventional radio interface are awakened when the sensor node is addressed, so that when a wake-up pattern is recognized, data exchange can occur. Of course, the WuR is always ready to detect and identify wake-up signals generated by the coordinating nodes in the WSN to maintain a low latency. To save power consumption and avoid waking up other nodes, the WuRs employ distinctive wake-up patterns and identifiers so that each node can be addressed individually. To perform this task, the WuRs are composed of an RF front-end and a digital baseband back-end, with a pattern recognizer or signal correlator used to identify the wake-up identifier. Power savings introduced by using WuRs are mostly observed in low-activity and low-average throughput applications, as the main radio interface is in sleep mode during a significant amount of time [4]. Due to its inherent simplicity, the on–off keying (OOK) modulation scheme is frequently the preferred option when designing a WuR. This scheme is equivalent to an amplitude-shift keying (ASK) of two levels, and it consists of a sinusoidal signal modulated with an on-and-off unipolar binary signal. Very simple architecture is needed to demodulate the OOK signal. At the same time, since very simple architecture is employed, meeting high performance metrics is a design challenge, and the design of high-sensitivity, high data rate, low-power-consumption WuRs is not a straightforward procedure. Although significant power savings are achieved with a low impact in latency, this advantage comes at the expense of somewhat higher system complexity, size and cost for the WSN node [5]. In addition, the limited power consumption characterizing a WuR also limits the achievable sensitivity and selectivity, so conventional receivers are clearly superior to WuRs in these terms. This means that a higher transmitting power may be needed to reach a WuR when compared to the power needed to reach a conventional receiver for the same distance. However, in densely populated networks the priority is to reduce the cost-per-node as much as possible, so that the sensitivity/power consumption trade-off is not a design focus. Therefore, system integration, circuit area, assembly costs, bill of materials and calibration strategies are the main concern in these kinds of scenarios.

**Figure 2:**
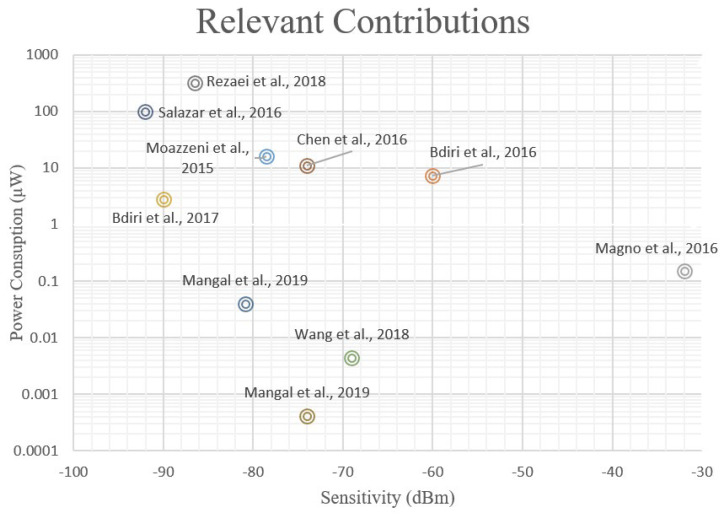
Representation of some of the most relevant state-of-the-art contributions in WuR design. Power consumption and sensitivity are the two most relevant parameters for WSN applications and, thus, the contributions are compared by means of these two metrics.

Nowadays, the integration of WuRs in commercial applications and consumer electronics is increasing, with industry and academia spending more, and more design effort in reducing the cost-per-node and power consumption and increasing sensitivity. This can be seen in several state-of-the-art works, since some authors have reported circuits achieving very high sensitivities while operating in the nW range. In this sense, the performance of some of the most relevant contributions in the literature are depicted in Figure 2; the *X*-axis refers to the sensitivity and the *Y*-axis covers the power consumption. In comparison with conventional receivers, in which the power consumption is frequently in the order of mW, a sub-nW power consumption is clearly remarkable. The advantage of these solutions is that the circuits can be powered by coin-sized batteries or even employ energy-harvesting strategies and are able to perform continuous channel listening for years. The approach adopted in [6] is an example of this idea, where a battery-less WuR with energy-harvesting techniques is implemented to produce a stable DC supply from the RF signals in the channel, to feed the different components of the system. Other works [1] define WuRs able to react to certain events or environmental conditions and awake the MCU as a consequence. Thanks to the implementation of low-power sensors for this task and because the systems are less prone to generating false wakeups, these solutions can result in a more efficient operation and a lower power consumption.

Many WuRs are based on the principle of envelope detection, consisting of a simple rectifier, and avoiding the use of local oscillators and phase-locked loops (PLLs), which leaves room for remarkable power savings at the cost of a limited sensitivity [1]. In Figure 2, it is clearly seen that a trade-off between power consumption and sensitivity exists in WuR implementations. There is a frontier delimited by an imaginary line at the left-hand side of the figure, going from about 1 mW of power consumption to –75 dBm of sensitivity. Certainly, architecture selection plays a critical role when designing a WuR since some topologies lean toward higher power savings at the expense of sensitivity, and vice versa. One of the most significant differences lies in the use of active components such as amplifiers, mixers, or oscillators. When active RF components are used, the sensitivity is boosted, as well as the power consumption. On the other hand, it is possible to implement sub-nW WuRs if the power-hungry RF active components are kept to a minimum, or if they are carefully designed to present very low power consumption, thus improving the power consumption; however, the sensitivity is then limited to about –80 dBm.

In previous literature, different architectures provide sensitivity optimizations without major reductions in circuit area and costs-per-node, which are of utmost importance for system integration and low-cost densely populated networks. The aim of this work is to address these issues, presenting two different WuR implementations based on the tuned-RF architecture. The first circuit is based on [7], it employs a feedback amplifier and a band-pass filter to perform the envelope detection task. This circuit is developed as a proof-of-concept to replicate the results in [7]. The circuit achieves performance in line with most of the state-of-the-art solutions; however, it can be improved. Our main contribution is the introduction of the current-reuse feedback amplifier presented in [8] in the circuit proposed in [7], to improve the sensitivity, power consumption and area simultaneously. Therefore, in the second implementation of the WuR, the complex two-stage feedback amplifier is substituted by a feedback current-reuse topology. Both designs are presented from the schematic to the layout stage and are implemented in a low-cost standard CMOS process.

The remainder of this article is organized as follows: firstly, Section 2 offers a review of the existing WuR architectures and mentions some of the most relevant state-of-the-art contributions in each category. In Section 3, we present the development of two different WuRs in a low-cost CMOS commercial process. The performance of the proposed solutions and a comparison with the most relevant state-of-the-art solutions are shown in Section 4, and conclusions of this work are drawn in Section 5.

## 2. Wake-Up Receiver Architectures and State of the Art

A wide variety of topologies can be adopted when designing a WuR, as seen in 3. Although Zero- and Low-IF architectures are most frequently used in conventional receivers [9,10,11,12,13] they generally lead to high sensitivity, but have a power consumption that makes them unsuitable for low-power applications. Therefore, different solutions have been explored to reduce the power consumption of the receiver at the cost of reducing the sensitivity.

**Figure 3:**
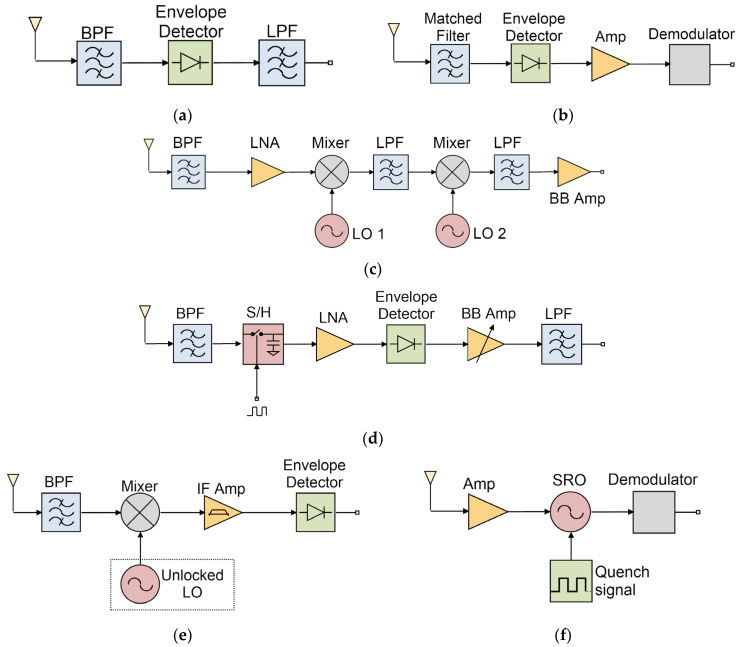
WuR topologies: envelope detector (**a**); matched-filter (**b**); superheterodyne (**c**); sub-sampling (**d**); uncertain-IF (**e**); and super-regenerative (**f**); and architectures.

On the one hand, the simplest and most-used technique in the field of WuRs is radiofrequency envelope detection (RFED), shown in Figure 3a. With this approach the received signal is demodulated directly to base-band, avoiding the need for mixers and local oscillators (LOs), and potentially achieving very low power consumption [5]. Nevertheless, due to the inherent nonlinearity and wideband characteristics of the envelope detection process, RFED architectures are characterized by a high noise figure and low interference resilience when compared to other WuR architectures. Thus, the sensitivity these solutions can achieve is limited [13]. A common solution to improve the WuR’s sensitivity consists of placing an LNA before the envelope detector to boost the signal-to-noise ratio (SNR), this architecture is known as tuned RF (TRF). A high gain is required from the LNA to reduce the contribution of the noise figure (NF) of the envelope detector, leading to a trade-off between sensitivity and power dissipation [4]. To improve the circuit’s interference resilience, high-Q off-chip components can be used to implement the input filter [13]. Due to its simple architecture, and because many works prioritize power consumption over sensitivity, the RFED is one of the most frequent topologies in the literature [1,6,14,15,16,17,18]. In [6], a battery-less energy-harvesting WuR is demonstrated by implementing a passive RFED-based architecture. The front end uses no external power source since the receiver converts the incident RF signal in a stable DC voltage to supply the different building blocks. In addition, various contributions [14,15,16] have reported RFED-based WuRs with very high sensitivities and a power consumption in the order of a few nW, by adding a large passive gain before the envelope detector. A sub-nW WuR achieving a sensitivity of −79 dBm is presented in [17], by implementing multi-stage passive energy detectors as well as a high-Q input matching network. However, these contributions exploit the latency–sensitivity trade-off by relaxing latency constraints to achieve such high WuR sensitivities [17].

A less common approach is the matched-filter architecture, employing widespread spectrum techniques to relax the dependence on the received signal strength and the issues related to in-band interferers. With the implementation of matched filters, the incoming signal spectrum is efficiently recovered in the receiver. A block diagram of this topology is presented in Figure 3b. These structures usually achieve low power consumption and high selectivity at the expense of sensitivity, caused by a high insertion loss due to the input matched filter [18]. An innovative code-domain matched filter is implemented in [19] to design a WuR with a sensitivity of –80.9 dBm and a power consumption of only 40 nW. The main receiver features a gate-biased envelope detector, followed by a current-reuse amplifier and an analog correlator. The analog correlator is composed of VCOs, delay lines and DACs, and a 4-phase filter is used to suppress VCO frequency and associated harmonics. The output of the correlator is then fed to a comparator to identify the wake-up signal. The conventional superheterodyne (SH) architecture is a well-known, widely explored solution in the field of low-power receiver design. As shown in Figure 3c, the architecture employs an LNA and one or more mixers [20], followed by an IF amplifier to boost the SNR, and an ADC for digital base-band (DBB) signal processing. Since performing signal amplification at IF instead of RF is more power-efficient, such approaches often obviate the inclusion of an LNA to reduce power consumption at the expense of dealing with a higher NF. Following this approach can be advantageous in terms of sensitivity and interferer resilience, mainly because it is possible to design highly selective, low-power IF filters to reduce the impact of noise and blockers [13]. Generally, the SH receiver architecture yields a very high selectivity and overall performance compared to other solutions, but it presents a severe disadvantage. Due to the frequency synthesizers required to generate the mixing frequencies of the LO, the SH topology may present excessive power consumption, and thus, mixer-based architectures are only seen in applications where a power consumption over the range of tens of μW are acceptable. The superheterodyne architecture is possibly the most challenging approach to achieve an adequate performance in WuR design, mostly because of its complexity and the narrow margin to obtain power savings when compared to other architectures [4]. An example of an SH-based WuR is reported in [21], targeted for the band of 2.4-GHz with a high interference resilience. The receiver is designed for OOK-modulated signals, featuring a dual-IF N-path architecture with multiple inter-stage VGAs. The receiver provides a high sensitivity of –97 dBm at a data rate of 10 kbps, but the power consumption is as high as 99 μW.

The sub-sampling architecture is similar to the superheterodyne receiver, but in this case, the mixers are substituted by sample-and-hold (S/H) circuits [18], as depicted in Figure 3d. In this structure, the input signal is filtered and then converted to IF by the S/H circuit, which is followed by an IF amplifier and an envelope detector. In these receivers the RF LO is replaced by an accurate low-frequency LO, resulting in significant power savings [21]. Some works may include an RF LNA to improve receiver sensitivity, but then the power dissipation will be dominated by this element. One of the advantages of the sub-sampling architecture is that the designer has a certain degree of freedom when selecting the intermediate frequency (IF), since the sampling process results in the appearance of multiple signal replicas in the frequency domain [18]. Sub-sampling-based WuRs can achieve better sensitivity than RFED-based WuRs, but also at the cost of increased power consumption. A 2.4-GHz WuR based on the sub-sampling architecture can be found in [22]. The WuR is severely duty cycled to achieve the small power consumption of 7 μW, it achieves a remarkable sensitivity of –80 dBm and a reaction time of 30 ms. In [23], a dual-mode receiver with a sensitivity of –78.5 dBm and –75 dBm with a power consumption of 16.4 μW and 22.9 μW for the two modes (10 and 200 kbps data rates, respectively) is reported. The approach employs a two-stage differential S/H circuit followed by an LNA, an IF amplifier, and a conventional NMOS-based envelope detector.

One of the problems of having an LO is the need to include a PLL to generate the fixed, stable frequency required for signal down-conversion, which is a very expensive element in terms of power consumption. Similarly, as previously mentioned, the power overhead attributed to front-end LNAs can be quite significant. The uncertain-IF architecture, shown in Figure 3e, is aimed to address these two issues. This receiver structure implements no LNA and presents an IF that can vary in a delimited range, which is defined by an LO without PLL [18]. By doing so, the power consumption needed to amplify the signal at IF is reduced, too. In this case, since the signal down-conversion process covers a wide frequency range, uncertain-IF receivers are very vulnerable to interferers and, therefore, they usually require an external high-Q input filter [24]. In addition, this architecture can present high sensitivity, but it is limited due to the wide IF range the receiver must cover to be able to manage LO uncertainty, and the associated noise [21]. Nevertheless, the uncertain-IF architecture can be exploited to obtain sensitivities close to the SH architectures, while consuming less power. An example of an uncertain-IF based WuR for the UHF 408-MHz frequency band can be found in [25]. The receiver achieves a sensitivity of –55 dBm at 50 kbps with a power consumption of 100 μW. The architecture employs a SAW filter at the input to improve selectivity, a mixer, and a multistage IF amplifier, followed by an envelope detection stage.

A unique solution to avoid the need to include PLLs is to use a tuned oscillator as a frequency-to-amplitude conversion block to extract FSK-modulated data from the incoming signal [26]. This is the principle behind the injection-lock architecture [27,28,29]. If the carrier frequency of the input signal is close to the LO frequency, coupling between both signals occurs and a single frequency appears at the output. This effect is called injection locking. However, if the carrier frequency of the incoming signal is not close enough to the LO frequency, the LO is perturbed, but there is no coupling between the signals, and the system generates two different tones. This is the effect of injection pulling. One of the drawbacks of using this technique is that the injection-lock receiver relies significantly on the input signal strength, and a trade-off between receiver sensitivity and power dissipation in RF amplifiers has to be considered by the designer [29]. A differential injection-lock-based receiver front-end incorporating an external loop antenna is developed in [29], operating at 433 MHz with a power consumption of 54 μW and a low-power of 11 μW. The receiver shows a sensitivity of –80/–78/–77 dBm under OOK/FSK/PSK modulation schemes at a data rate of 200 kbps. Additionally, an injection-locking digitally controlled oscillator is introduced in [28], reporting a sensitivity of –62 dBm for a data rate of 312 kbps, operating at 80 MHz with a power consumption of 45 μW.

As in the injection-lock topology, the super-regenerative oscillator (SRO) idea is another approach in which the generated oscillations are dependent on the input signal strength. The architecture, as shown in Figure 3f, is characterized by an RF oscillator changing periodically between stable and unstable states, and it is controlled by a low-frequency quench oscillator [30]. When the main oscillator is in stable-mode operation, the generated output signal amplitude is linearly proportional to the input signal amplitude. One distinctive characteristic of SRO-based WuRs is that the bias current of the oscillator is dynamically modulated to change between the two states. This can lead to a more selective bandpass filter than in injection-lock receivers, where an oscillator with a constant bias current is used [24]. It is, therefore, theoretically possible to achieve a better performance with an SRO-based WuR than with an injection-lock architecture in similar conditions. In contrast to the injection-lock and uncertain-IF architectures, an SRO-based WuR can receive OOK-, FSK- and ASK-modulated signals. Additionally, SRO-based WuRs are generally superior to SH-based WuRs due to the existence of positive feedback, which enhances receiver sensitivity and reduces power consumption [24]. The limitations of the SRO architecture are the excessive spurious emissions that do not meet some standard regulations, and the distortion introduced to the output signal. The latter, however, is less of a concern for amplitude modulation schemes. Hence, although the SRO-based WuRs often suffer from poor selectivity, the topology is employed in scenarios such as key-less remote transmitters and AM receivers [24]. While the SRO architecture can provide decent performance even at a sub-100 μW range, it suffers from some fundamental challenges. The SRO architecture presents the great disadvantage of requiring multiple calibration schemes to guarantee proper performance [24]. In addition, unknown frequency offsets between the desired channel and its operating frequency can be produced by the amplitude variation at the SRO’s output. Finally, during the implementation of an SRO-based receiver, the designer must consider the existing trade-offs between sensitivity and power consumption, and back-radiated signal power. In [31], the authors propose an SRO-based WuR with a self-quench circuit and a technique to prevent oscillations when no carrier is present. This solution employs PWM wake-up codes and low-power decoding, achieving a sensitivity of –63.8 dBm and a power consumption of 20 μW, operating at the 28-MHz human-body communications (HBC) frequency band. A binary search algorithm is used in [24] for calibration of the quench signal and the receiver’s operating frequency. A sensitivity of –86.5 dBm and –101.5 dBm is reported for data rates of 1000 kbps and 31.25 kbps, respectively. The system operates in the 902–928 MHz ISM band with a power consumption of 320 μW. In addition, a high-performance SRO-based WuR with a power consumption of only 1 μW and a sensitivity of –90 dBm has been reported [32].

Considering all the alternatives presented, the envelope detection approach is the architecture that achieves the lowest power consumption [5,19]. This feature comes at the cost of limited sensitivity, making the RFED technique suitable in WSNs where the nodes are close to each other. In applications with scattered nodes, alternative structures may produce more appropriate results due to the need for higher sensitivity. Otherwise, an increment in transmitted signal power would be required to reach the nodes and overcome the poor sensitivity characterizing the sensors’ RF interface. The tuned-RF strategy can solve this issue by introducing better sensitivity at the expense of slightly higher power consumption. Since many works include off-chip components to improve receiver performance, namely power consumption, sensitivity and selectivity, WuR integration is another challenge [21]. Many of these contributions show high power efficiency, but they usually suffer from a poor selectivity and high power dissipation, making them less suitable for densely populated WSN scenarios and applications requiring a power consumption under 100 μW [21]. As mentioned before, some WuR implementations use high-Q external resonators for narrowband filtering at RF, or to serve as accurate time-base references for the LO [21]. Nevertheless, the inclusion of these bulky and costly elements yields a more expensive bill of materials and assembly costs per device, which may be incompatible with low-cost WSNs [21]. Therefore, we avoid the use of this external circuitry to maintain a high level of integrability.

## 3. CMOS-Integrated WuR Design with a Feedback Configuration and Current-Reuse Technique

The implementation of the WuR is depicted schematically in Figure 1, with a target frequency of 868 MHz corresponding to the European definition of the industrial, scientific, and medical (ISM) band. Since the tuned-RF architecture is selected to implement the WuR, the OOK scheme is better suited as a modulation scheme for the wake-up signal. In the block diagram shown in Figure 1, the wake-up signal is received by the sensor while it is listening to the ISM channel. Node A represents the OOK signal at the WuR’s input, whereas at node B the wake-up signal’s envelope is obtained. The signal at node B contains the wake-up pattern or signature to be demodulated by the signal correlator. This element operates in the frequency range of 15 kHz to 150 kHz, and its main purpose is to recognize the wake-up pattern. If it is recognized, then an interruption is generated so that the main MCU is awakened, and conventional communications can occur. The T/R switch is set by the MCU and allows two possible paths for the input signal: a path to receive the wake-up signal when the sensor is in low-power mode, and a path for the conventional radio communications.

The wake-up signal used in this configuration is obtained from a low frequency sequence (0.5 to 4 kbit/s) modulated with a high frequency carrier (15 kHz to 150 kHz), which is then modulated again with a carrier of 868 MHz [3]. With this approach, the same antenna can be used for conventional communications and wake-up signaling. With this approach, a circuit as low-cost and low-power as the AS3933 can be employed as a signal correlator [13].

### 3.1. Tuned RFED WuR with a Feedback Pre-Amplifier

**Figure 4:**
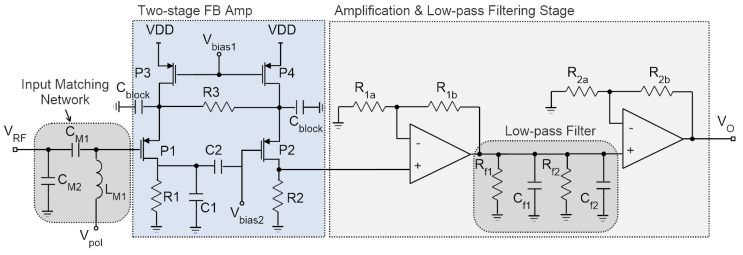
RFED WuR with a feedback amplifier stage [7].

The architecture employed to implement the WuR is based on the tuned-RF topology, so that the circuit is composed of an input matching network, a rectifier, and a low-pass filter. Some improvements can be applied to the basic RFED WuR scheme by adding a two-stage pre-amplifier to increase the sensitivity of the circuit, at the expense of slightly higher power consumption. This is the principle behind the tuned-RF architecture presented in Section 2. Moreover, this element can be implemented in a feedback configuration, as introduced by [7], boosting the WuR’s sensitivity with very low power consumption. The circuit implementation is shown in Figure 4, based on the work proposed by [7]. The design consists of two different blocks: the first stage is formed by an input-matching network, a low-power amplifier, a band-pass filter and a second ultra-low power amplifier; the second stage is composed of two base-band amplifiers and a low-pass filter.

**Figure 5:**
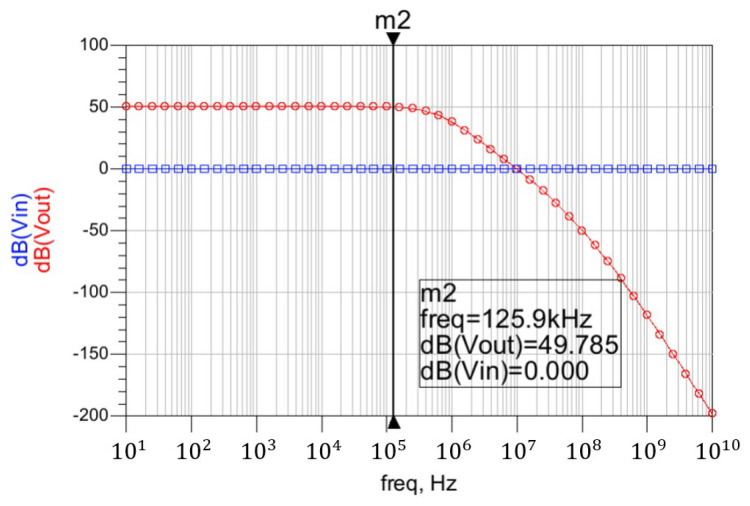
Simulation results of the pre-amplifier and the filter.

The feedback amplifier used in the first stage is composed by PMOS transistors, since they present lower flicker noise than NMOS transistors. The first amplification is performed by the common-source transistor P1, biased in weak inversion and drawing a current of 2.95 μA, which simultaneously performs as an RF detector and baseband LNA [7]. An RF suppression filter is formed by R1 and C1, and the MOS transistor P2 enhances the low frequency gain thanks to the feedback path opened through R3. The capacitor C_block_ sets the low cut-off frequency, whilst R1 and C1 adjust the upper cut-off frequency. This combination has a band-pass effect, performing the first step of the envelope detection task. The second common-source amplifier is formed by the PMOS transistor P2, biased with a drain current of 2.02 μA. The PMOS transistor P3, acts as the load of P1, and the same applies to P4 and P2. The amplifiers operate with a V_DD_ = 1 V, V_bias1_ = 0.01 V, V_bias2_ = 0.57 V and V_pol_ = 0.05 V, to maintain low power consumption. The second stage is composed by two operational amplifiers in a feedback non-inverting configuration, with a gain of 25 dB per stage and a second-order low-pass filter. The bandwidth of this stage has been designed to cover from 100 kHz to 1 MHz to obtain the OOK input signal’s envelope, as shown in Figure 5.

**Figure 6:**
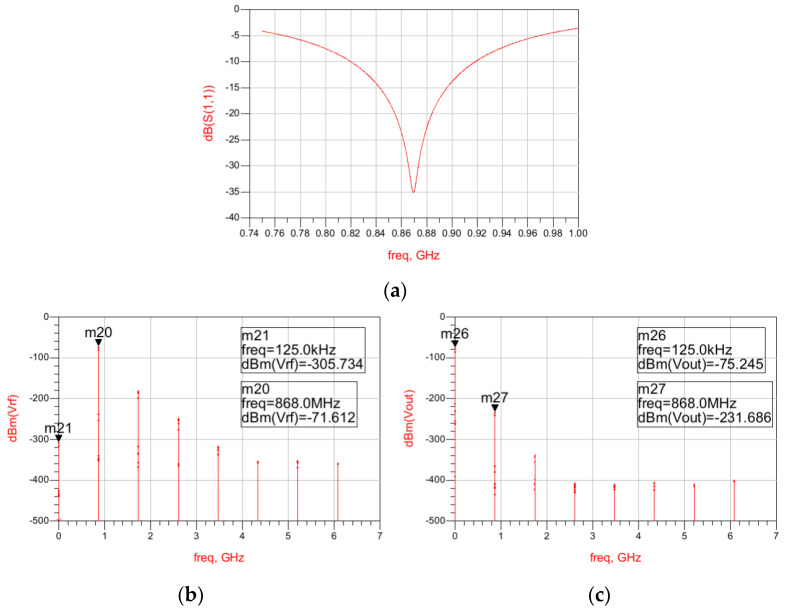
Input Return Loss simulation results of the feedback WuR with amplification and low-pass filtering stages (**a**) and harmonic balance simulation results of the WuR for the RF input signal (**b**) and the WuR’s output (**c**).

The results regarding the input matching of the full circuit are shown in Figure 6a, showing that an Input Return Loss better than 20 dB is obtained at 868 MHz in simulations. To determine the WuR’s sensitivity, a harmonic balance simulation is needed to verify the output power of the WuR. The harmonic balance simulation results of the complete WuR are depicted in Figure 6b,c, where we are using an AM input signal of –71.6 dBm and 868 MHz plus two tones (868.875 MHz and 868.125 MHz) of –75 dBm each. Considering that the AS3933 needs at least 113.15 µV_p_ to be able to recognize the pattern, a minimum input power of –63.2 dBm is required to be able to detect the WuR signal.

**Figure 7:**
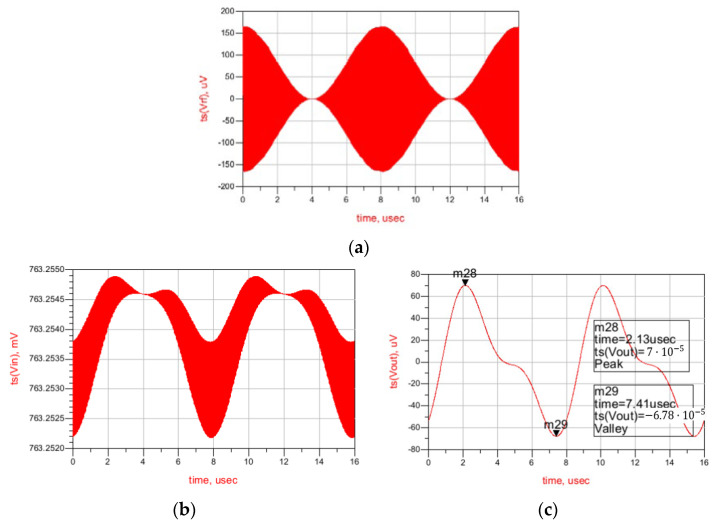
Time-domain simulation results of the WuR with amplification and low-pass filtering stages. AM input signal (V_rf_) (**a**), the output of the first stage (V_in_) (**b**), and the output of the second stage (V_out_) (**c**).

Finally, a time-domain simulation is presented in Figure 7, showing the AM input signal (V_rf_), the output of the first stage (V_in_), and the output of the second stage (V_out_). As shown, the signal at the first stage’s output contains the unfiltered envelope of the wake-up pattern, and after the second-stage filtering the output is completely clean. The value of the feedback resistors, as well as the components of the low-pass filter used, are given in Table 1. The complete stage presents a gain of 50 dB in simulations, with a power consumption of only 1.8 μW. The simulation results show a sensitivity of –63.2 dBm for the complete WuR, with a power consumption of 6.77 μW, concluding that this circuit achieves improved sensitivity and power consumption in comparison with the previously developed solutions.

**Figure 8:**
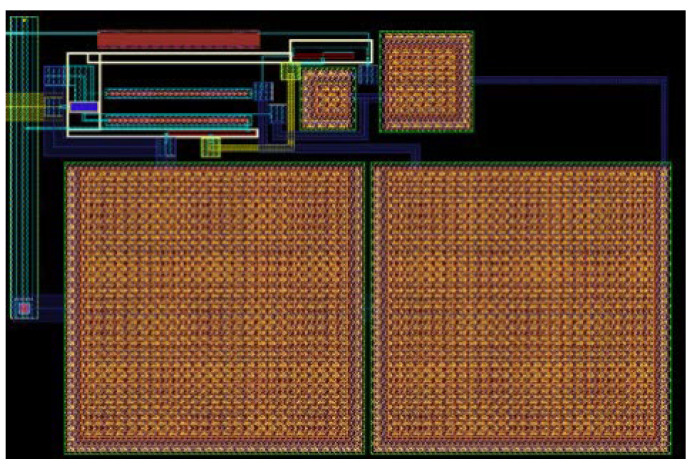
Layout of the two-stage feedback amplifier, total size of 230 × 160 μm^2^.

This design is implemented to operate from a 1-V DC supply with all the values given in Table 1. The layout of the first stage is presented in Figure 8, with a total size of 230 × 160 μm^2^. As seen, the two DC block capacitors are the bulkiest components in this implementation. The total size of the complete layout, including the op-amps and the second-stage low-pass filters, is 398 × 266 μm^2^.

### 3.2. Tuned RFED WuR with Current-Reuse Pre-Amplifier

**Figure 9:**
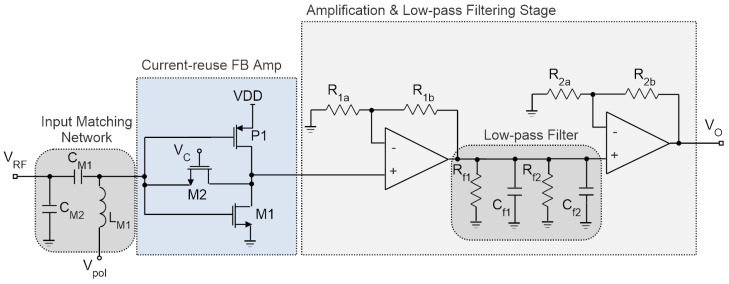
RFED WuR with current-reuse amplifier [8].

A possible improvement of the circuit presented in Figure 4 is to adopt a current-reuse approach to reduce the power consumption in the feedback amplifier of the first stage. In addition, by adopting the current-reuse technique, the current mirror formed by P3 and P4 can be eliminated and all the passives (R1 to R3 and C1, C2 and C_block_) can be simplified. The first-stage feedback amplifier with current-reuse is an inverter in a feeback configuration, and is depicted in Figure 9 [8].

The enhancement introduced by this topology is mainly due to the addition of the PMOS transistor’s transconductance to the equivalent g_m_ of the circuit, as shown in (1), where *g_mN_* and *g_mP_* are the NMOS and PMOS transconductances, respectively, and *R_F_* is the value of the feedback resistor. The PMOS P1 reuses the biasing current of the NMOS transistor M1, with a value of 1.83 μA from a 1-V DC supply. Transistor M2 is used in the feedback path since a high resistance is required to maintain a low NF, also achieving high g_m_-boosting for improved AC gain and a reasonable power consumption [8]. By using M2 instead of a conventional resistor the circuit avoids the large footprint and resistance tolerances associated with integrated resistors, while achieving a very small area. The only drawback of this approach is the need for an additional DC control voltage (V_C_), which is set to 0.72 V. Likewise, V_pol_ is set to 0.45 V to bias the gate of M1 and P1 with a reasonable performance. To obtain high resistance, transistor M2 is a long-length, narrow device with a size of (80 nm/3 μm). Since it presents a feedback configuration, the input impedance of the circuit is given by (2), where *A_v_* represents the circuit AC gain. The AC gain increment ensures a high bandwidth, given as (3), since it relaxes the contribution of the parasitic capacitances due to M1 and P1. In this equation, *C_gsN_* and *C_gsP_* refer to the NMOS and PMOS gate-source capacitance, respectively. In this case, the band-pass filter in the feeback amplifier is eliminated, as the filter in the second stage is enough to obtain the input signal’s envelope. The advantage of this topology is the compact size and low power consumption achievable thanks to the implementation with only three MOSFETs.

(1) $$\left|A_{v}\right|\approx \left(g_{mN}+g_{mP}\right)\cdot R_{F}$$

(2) $$Z_{in}\approx \frac{R_{F}}{1+\left|A_{v}\right|}$$

(3) $$BW_{3\mathrm{dB}}\approx \frac{1+\left|A_{v}\right|}{R_{F}\left(C_{gsN}+C_{gsP}\right)}$$

**Figure 10:**
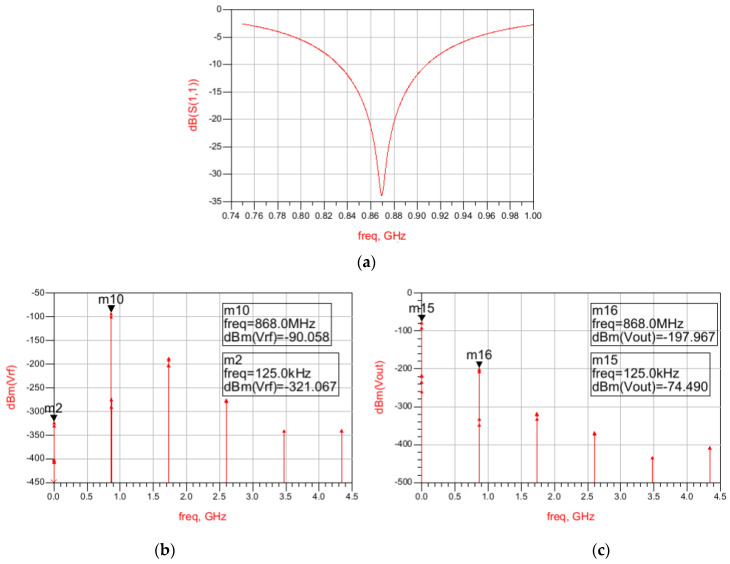
Input Return Loss simulation results of the current-reuse feedback WuR with amplification and low-pass filtering stages and harmonic balance simulation results. Input Return Loss of the circuit (**a**), harmonic balance AM input signal (**b**), and WuR’s output signal (**c**).

As shown in Figure 10a, an Input Return Loss better than 20 dB is obtained at 868 MHz in simulations for the full circuit including the second-stage amplifier and filter. In addition, the harmonic balance simulation is presented in Figure 10c. In this case, we are using an AM input signal of –90 dBm and 868 MHz, plus two tones of –95 dBm each, resulting in a sensitivity of –75 dBm. 

**Figure 11:**
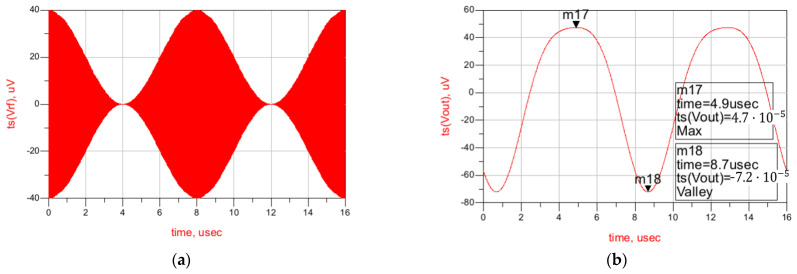
Time-domain simulation results of the current-reuse feedback WuR with amplification and low-pass filtering stages. Input (**a**) and output signals (**b**).

The results regarding the time-domain simulation are shown in Figure 11. As seen, the signal is effectively filtered, and at the output of the WuR signal is completely clean.

**Figure 12:**
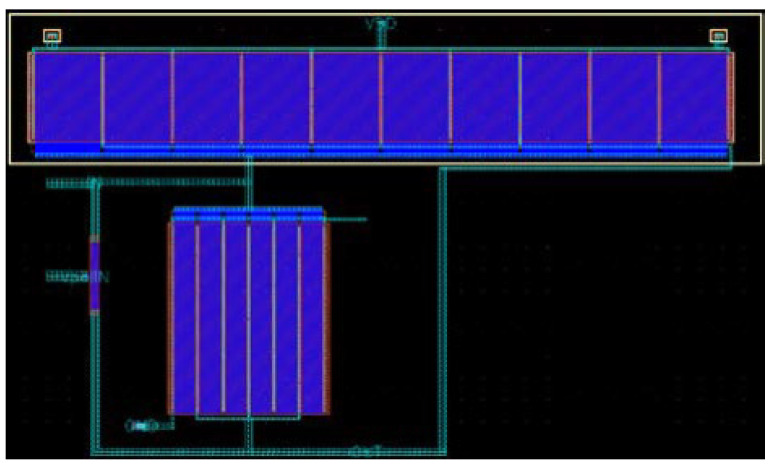
Layout of the current-reuse feedback amplifier used in the WuR.

The layout of the current-reuse feedback amplifier, shown in Figure 12, occupies an area of only 20 × 35 μm^2^ with a power consumption of 1.83 μW. The complete layout, including the op-amps and low-pass filters, results in a size of 262 × 262 μm^2^. The circuit is almost ten times smaller than the previously developed feedback amplifier, since there are no passive elements in the structure. All the elements forming the second stage have their values presented in Table 2. The full circuit simulations reveal an input return loss of 32.5 dB, with a sensitivity of –75 dBm and a power consumption of only 3.63 μW.

## 4. Overview and Discussion

**Figure 13:**
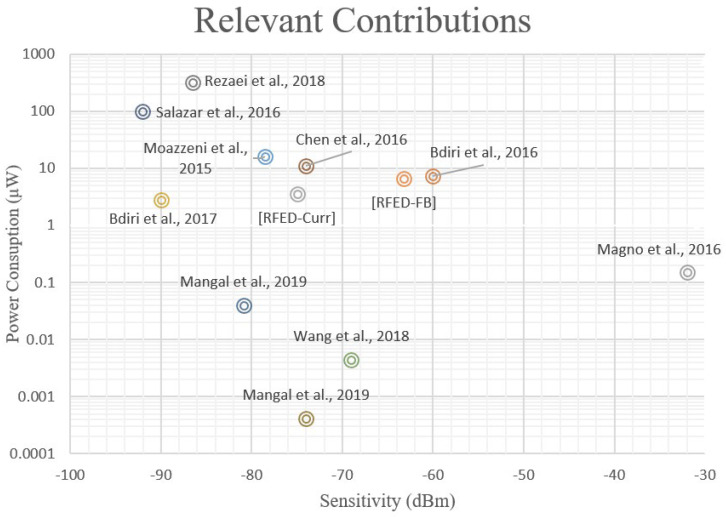
Representation of the performance of the proposed contributions along with some of the most relevant state-of-the-art works involving WuR design.

Two structures based on the tuned-RF principle have been explored. To improve the WuR’s sensitivity and power consumption simultaneously, a structure formed by a feedback amplifier and a band-pass filter, followed by two op-amps and a low-pass filter, is studied. This circuit obtains a sensitivity of –63.2 dBm, with a power consumption of only 6.77 μW and a total size of 398 × 266 μm^2^, of which only 230 × 160 μm^2^ are occupied by the feedback pre-amplifier. Thus, with this approach, the sensitivity and power consumption of the WuR are simultaneously improved. The second design presented utilizes a current-reuse feedback amplifier to further reduce the power consumption and die footprint. This approach gives a remarkable sensitivity of –75 dBm in simulations with a power consumption of only 3.63 μW and a die footprint as low as 262 × 262 μm^2^, of which only 20 × 35 μm^2^ are occupied by the current-reuse feedback amplifier in the first stage. That is, the area of the first stage is reduced from 36,800 μm^2^ to 700 μm^2^, simultaneously improving the WuR’s sensitivity and power consumption. The tuned-RF WuR with current-reuse pre-amplifier achieves one of the best trade-offs between performance and die footprint of all the explored solutions. A visual comparison between our main contributions and some of the most relevant state-of-the-art solutions in the field of WuR design, in terms of power consumption and sensitivity, is presented in Figure 13. The acronym RFED-FB refers to the tuned-RF WuR with a feedback topology, and RFED-Curr refers to the current-reuse circuit presented in this work. Note that only sensitivity and power consumption are considered in the figure, and the die footprint is not included. A comparison of circuit performance is given in Table 3 with a higher level of detail. In the table, the value used for the figure of merit (FoM_LAT_) is obtained from [13], and is defined in Equation (4). Again, in this equation, only sensitivity and power consumption are considered as the two most relevant performance parameters in WSN nodes. However, this definition of the FoM may not be as suitable for applications where the nodes present a high throughput. That is why we used the FoM_ARE_ in Equation (5) [3], to include the circuit area. As seen in Table 3 and in Figure 13, the RFED WuR with current-reuse feedback amplifier presents a better trade-off that the other proposed circuits.

The authors in [4] also adopt a tuned-RF architecture in the implementation of a WuR, with a remarkable performance. To reduce power consumption with low latency, a modified MAC protocol, along with enhanced duty-cycled listening, are used. In [13], an integrated transformer is used within an RFED WuR to obtain a passive gain of 25 dB achieving a very high sensitivity with a power consumption of only 4.5 nW. On the other hand, the work in [21] is an example of an uncertain-IF WuR. In this proposal, the power consumption is 99 μW while achieving an outstanding sensitivity of –97 dBm. An example of the injection-lock architecture can be found in [29]. In this article, an external loop antenna is presented, and two operation modes are implemented to obtain high sensitivity and low power consumption. The WuR shown in [24] is based on the SRO strategy, and therefore, is characterized by the highest power consumption of all the explored solutions. A fully integrated WuR is proposed in [17]. The circuit is composed of 40-stage gate-biased self-mixers and matched filters with DC offset cancellation, achieving a power consumption of about 1 nW and sensitivity of –74 dBm. The authors of [19] present a WuR based on a code-domain matched-filtering strategy, where a continuous time analog correlator is employed as pattern recognizer, reporting a very high performance WuR. The work in [2] proposes the implementation of an RFED WuR with off-chip components; the envelope detector is composed by Schottky diodes and the AS3933 circuit is used as correlator. Finally, another RFED WuR in a PCB fashion is presented in [33] using the TI CC430F5147 SoC, which implements an MCU and a transceiver. This solution employs two antennas to improve the accuracy instead of sharing the path by using a T/R Switch, as we did. It is seen that the developed WuR with a current-reuse feedback amplifier is superior to the proposals in [2,23,24,29,33] in terms of the sensitivity and power consumption trade-off. If we include the area efficiency in the FoM as indicated by Equation (5), the current-reuse feedback amplifier is clearly superior to all the explored solutions but [17,19], which are superior both in sensitivity and power consumption. Thus, we can conclude that the current-reuse feedback WuR presented in this work shows a performance in line with most of the state-of-the-art contributions and is superior in terms of area use. This fact makes the circuit especially suitable for circuits and application scenarios where a high integrability is required.

(4) $${\mathrm{FoM}}_{\mathrm{LAT}}\left(\mathrm{dB}\right)=-P_{SEN}\left(\mathrm{dBm}\right)-P_{DC}\left(\mathrm{dBm}\right)-60$$

(5) $${\mathrm{FoM}}_{\mathrm{ARE}}\left(\mathrm{dB}\right)=-P_{SEN}\left(\mathrm{dBm}\right)-P_{DC}\left(\mathrm{dBm}\right)-10*\log\left(area\left({\mu m}^{2}\right)\right)$$

## 5. Conclusions

The design of two WuRs based on the tuned-RF principle operating in the 868-MHz ISM band has been presented. The focus of our proposals is centered on area reduction to provide high integrability and maintain a low cost-per-node. In particular, we have presented a design based on [7] and improved the area use of the circuit by adding a current-reuse approach as in [8]. These characteristics result in an easier implementation of sensor nodes in low-cost IoT applications. The discussed alternatives show a performance in line with most of the state-of-the-art contributions, with a very low die footprint, making the topologies attractive in situations where a high integrability and low cost-per-node is pursued. The first solution is composed of a feedback amplifier, two op-amps plus a low-pass filter. The circuit achieves a sensitivity of –63.2 dBm with a power consumption of 6.77 µA and an area as low as 398 × 266 µm^2^. The second design is aimed at improving the power consumption and area simultaneously by using a current-reuse feedback amplifier. With this technique the power consumption is halved (resulting in 3.63 µA) and the resulting circuit area is as low as 262 × 262 µm^2^. Thanks to the nature of the circuit, the sensitivity is improved to –75 dBm. This latter proposal is particularly suitable in applications where a fully integrated WuR is desired, providing a reasonable sensitivity with low power consumption and a very low die footprint, therefore facilitating the integration with other components of the WSN node. A thorough discussion of the most relevant state-of-the-art solutions has been presented, too, and the two developed solutions are compared to the most relevant contributions available in the literature. Two different definitions of the FoM are presented, with one valuing mainly the sensitivity and power consumption, and another considering the circuit’s area as well. In this sense, it is shown that the current-reuse feedback WuR is superior to most of the explored solutions and achieves one of the best trade-offs between area and performance.

## Figures and Tables

**Table 1:** Summary of component values for the tuned RFED with feedback amplifier WuR.

Parameter	Value	Parameter	Value
P1	(200 μm/3 μm)	L_M1_	6.7 nH
P2	(1.6 μm/0.4 μm)	R1	90 kΩ
P3	(1 μm/3 μm)	R2	799 kΩ
P4	(2 μm/2 μm)	R3	250 kΩ
C1	1.4 pF	R_1a_, R_1b_	1 kΩ
C2	300 fF	R_2a_, R_2b_	17 kΩ
C_block_	250 nF	R_f1_, R_f2_	100 Ω
C_block2_	400 pF	V_bias1_	0.01 V
C_f1_, C_f2_	10 pF	V_bias2_	0.57 V
C_M1_	1.9 pF	V_pol_	0.05 V
C_M2_	840 fF	V_DD_	1 V

**Table 2:** Summary of component values for the tuned RFED with feedback amplifier WuR.

Parameter	Value	Parameter	Value
M1	(51 μm/0.97 μm)	R_1a_, R_1b_	1 kΩ
M2	(0.08 μm/3 μm)	R_2a_, R_2b_	17 kΩ
P1	(1 μm/3 μm)	R_f1_, R_f2_	100 Ω
C_f1_, C_f2_	10 pF	V_C_	0.72 V
C_M1_	1.1 pF	V_pol_	0.45 V
C_M2_	1.02 pF	V_DD_	1 V
L_M1_	4.26 nH		

**Table 3:** Performance summary of the explored RFED-based solutions.

WuR Topology	Frequency (GHz)	Sensitivity (dBm)	Power Consumption (μW)	Area (μm^2^)	FoM_LAT_ (dB)	FoM_ARE_ (dB)
RFED w/feedback amp.	0.868	–63.2	6.77	398 × 266	84.9	34.6
RFED w/current reuse	0.868	–75	3.63	262 × 262	99.4	51
[2] RFED	0.868	–60	7.5	37,000 × 22,000	81.2	−7.9
[33] RFED	0.868	–32/–55	0.152/1.2	--	70.2/84.2	--/--
[4] Tuned-RF	0.868	–90	2.8	46,300 × 24,500	115.5	25
[23] Sub-sampling	0.915	–78.5	16.4	1000 × 200	96.4	43.3
[13] RFED w/transformer	0.1135	–69	0.0045	(PCB)	122.5	--
[21] Uncertain-IF	2.4	–97/–92	99	360 × 160	107.0/102.0	54.4
[29] Injection-Lock	0.433	–80/–74	54/11	900 × 500	91.4/93.6	37.1
[24] SRO	0.915	−86.5	320	900 × 500	91.4	34.9
[17] Self-mixer + DLL	0.151/0.434/1.016	–79/–79.2/ –74	~0.00042	370 × 250	142.8/143/137.8	93.1/93.3/88.1
[19] CT-Analog Correlator	0.4508	–80.9	0.04	500 × 480	124.9	71

## Data Availability

Not applicable.

## Abbreviations

ADC	Analog-to-Digital Converter
ASK	Amplitude-Shift Keying
CMOS	Complementary Metal-Oxide Semiconductor
DAC	Digital-to-Analog Converter
DBB	Digital Baseband
DC	Direct Current
FoM_ARE_	Figure of Merit (Area)
FoM_LAT_	Figure of Merit (Latency)
FSK	Frequency-Shift Keying
HBC	Human-Body Communications
IF	Intermediate Frequency
IoE	Internet of Everything
IoT	Internet of Things
ISM	Industrial, Scientific, and Medical
LNA	Low-Noise Amplifier
LO	Local Oscillator
MAC	Medium-Access Control
MCU	Microcontroller Unit
MOSFET	Metal-Oxide Semiconductor Field-Effect Transistor
NF	Noise Figure
OOK	On–Off Keying
PLL	Phase-Locked Loop
PSK	Phase-Shift Keying
PWM	Pulse-Width Modulation
RF	Radiofrequency
RFED	Radiofrequency-Envelope Detection
SH	Superheterodyne
S/H	Sample-and-Hold
SoC	System-on-Chip
SRO	Super-regenerative Oscillator
SNR	Signal-to-Noise Ratio
T/R	Transmitter/Receiver
TRF	Tuned-RF
VCO	Voltage-Controlled Oscillator
VGA	Variable-Gain Amplifier
WuR	Wake-up Receiver
WSN	Wireless Sensor Network
